# Rare-Earth Oxide Nanoparticles: A New Weapon Against Multidrug-Resistant Pathogens with Potential Wound Healing Treatment

**DOI:** 10.3390/nano15241862

**Published:** 2025-12-11

**Authors:** Albert Donald Luong, Moorthy Maruthapandi, Aharon Gedanken, John H. T. Luong

**Affiliations:** 1 Innovative Wound Care (IWC), Fresno, CA 93710, USA; dluong.md@gmail.com; 2 Department of Chemistry, Bar-Ilan Institute for Nanotechnology and Advanced Materials, Bar-Ilan University, Ramat-Gan 52900, Israel; maruthapandimartin.m@gmail.com (M.M.); gedanken@biu.ac.il (A.G.); 3 School of Chemistry, University College Cork, T12 YN60 Cork, Ireland

**Keywords:** rare earth element oxides, antimicrobial, inhibition mechanism, cytotoxicity, production methods, green approach

## Abstract

Rare-earth oxide (REO) nanoparticles (NPs)—such as cerium (CeO_2_), samarium (Sm_2_O_3_), neodymium (Nd_2_O_3_), terbium (Tb_4_O_7_), and praseodymium (Pr_2_O_3_)—have demonstrated strong antimicrobial activity against multidrug-resistant bacteria. Their effectiveness is attributed to unique physicochemical properties, including oxygen vacancies and redox cycling, which facilitate the generation of reactive oxygen species (ROS) that damage microbial membranes and biomolecules. Additionally, electrostatic interactions with microbial surfaces and sustained ion release contribute to membrane disruption and long-term antimicrobial effects. REOs also inhibit bacterial enzymes, DNA, and protein synthesis, providing broad-spectrum activity against Gram-positive, Gram-negative, and fungal pathogens. However, dose-dependent cytotoxicity to mammalian cells—primarily due to excessive ROS generation—and nanoparticle aggregation in biological media remain challenges. Surface functionalization with polymers, peptides, or metal dopants (e.g., Ag, Zn, and Cu) can mitigate cytotoxicity and enhance selectivity. Scalable and sustainable synthesis remains a challenge due to high synthesis costs and scalability issues in industrial production. Green and biogenic routes using plant or microbial extracts can produce REOs at lower cost and with improved safety. Advanced continuous flow and microwave-assisted synthesis offer improved particle uniformity and production yields. Biomedical applications include antimicrobial coatings, wound dressings, and hybrid nanozyme systems for oxidative disinfection. However, comprehensive and intensive toxicological evaluations, along with regulatory frameworks, are required before clinical deployment.

## 1. Introduction

Rare-earth elements (REEs) comprise 17 elements: the 15 lanthanides (La–Lu), scandium (Sc), and yttrium (Y), which share similar chemical properties and often occur in the same ore deposits as the lanthanides. Although REEs are relatively abundant in the Earth’s crust, they are seldom found in economically viable concentrations. REEs are classified as light (LREEs: e.g., La, Ce, Nd) or heavy (HREEs: e.g., Dy, Er, Lu) based on their atomic structure, which influences their physical and chemical properties. REEs are valued for their magnetic, luminescent, and catalytic characteristics, enabling diverse applications in electronics, optoelectronics, magnets, energy technologies, glass, ceramics, defense, aerospace, and medicine [1] (Table 1). Not all REEs are in pure metal form, and several purification steps are needed to separate them. Promethium, the rarest, only occurs in trace quantities in natural materials with no long-lived or stable isotopes [2]. Notably, their roles in advanced devices for space, defense, and green technologies are well established, while their use in surgical lasers, MRI, and PET imaging is less recognized. Their unique optical and redox properties support applications in biosensing, medical imaging (e.g., Gd-based MRI contrast agents), cancer therapy (e.g., Lu-177, Ho, Y), and laser surgery (Er, Ho). Promethium (Pm) is distinct among REEs due to its radioactivity and extreme scarcity. All promethium isotopes are radioactive [3], with the most stable (Pm-145) having a half-life of 17.7 years. Natural promethium is virtually absent, and its synthesis requires nuclear reactors, limiting its use to specialized applications such as nuclear batteries for satellites and scientific research.

Beyond their antimicrobial properties, rare-earth elements exhibit antioxidant, regenerative, antidiabetic, and antitumor activities for important medical applications [4], as shown in Table 2. This review focuses on the production methods, antimicrobial mechanisms, and potential applications of selected REO-NPs as novel antimicrobial agents.

## 2. Mechanisms of Antimicrobial Properties

Rare-earth elements (REEs), including the lanthanides (La–Lu), scandium (Sc), and yttrium (Y), are increasingly recognized for their antimicrobial effects against bacteria and fungi. Although the exact mechanisms are still under investigation, they are known to disrupt microbial function through several key pathways:

1. Cell Membrane Disruption: REEs such as La^3+^, Ce^3+^, Gd^3+^, Y^3+^, and their oxides interact with negatively charged phospholipids, lipopolysaccharides, peptidoglycan of bacterial membranes, increasing permeability and causing leakage of cellular contents, ultimately leading to cell lysis [5]. Similarly, La^3+^, Ce^3+^, and Gd^3+^ bind to cell membranes, changing surface charge and causing permeability loss [6]. This is like the action of heavy metals like Ag^+^ and Cu^2+^. Considering electrostatic interaction, REE cations should have a strong affinity for the negatively charged phosphate backbone of DNA and RNA. This interaction can alter the structure and stability of nucleic acids (e.g., stabilizing certain DNA secondary structures or inducing DNA compaction) to disrupt the normal machinery of gene expression.
2. Ion Substitution: Due to their chemical similarity to essential metal ions (Ca^2+^, Mg^2+^, and Fe^3+^), REEs can replace these ions in enzyme active sites or membrane stabilization sites, resulting in enzyme inactivation and structural destabilization [5]. This disrupts critical cellular processes such as ATPase activity, transport, and cell wall biosynthesis. By occupying metal cofactor binding sites, REEs can also induce protein denaturation and inhibit key metabolic pathways such as glycolysis, the TCA cycle, and oxidative phosphorylation.
3. Generation of Reactive Oxygen Species (ROS): Ce^3+^/Ce^4+^ catalyze the formation of ROS (superoxide, hydroxyl radicals, and hydrogen peroxide), which induce oxidative damage to DNA, lipids, and proteins, leading to cell death [7,8,9]. CeO_2_ nanoparticles (Nanoceria) are considered a classical nanozyme with a high peroxidase activity-based nanozyme–H_2_O_2_ system that would be very efficient for bacterial disinfection. CeO_2_ NPs also possess high superoxide dismutase activity and antioxidant activity, which can act as a ROS scavenger [10].
4. Biofilm Disruption: REEs, especially La^3+^ and Ce^3+^, can interfere with quorum sensing and extracellular polymeric substance (EPS) production, reducing biofilm formation or promoting biofilm detachment [11].

The mechanisms and primary effects of some selected REEs are shown in Table 3.

## 3. Synthesis of Rare-Earth Oxide Nanoparticles (REO-NPs)

Rare-earth oxide nanoparticles (REO-NPs), such as CeO_2_, La_2_O_3_, and Nd_2_O_3_, are widely utilized in medicine and antimicrobial coatings. Several established methods enable the controlled synthesis of these nanoparticles.

1. Precipitation/Co-precipitation: Hydroxides or carbonates of rare-earth elements are precipitated from aqueous solutions and subsequently calcined to form oxides. This scalable method allows particle size control through adjustments in pH, temperature, and concentration. For example, homogeneous precipitation of CeO_2_ nanoparticles using ammonium cerium nitrate and urea yields particles around 8 nm [12].
2. Sol–Gel Method: Rare-earth alkoxides or nitrates undergo hydrolysis and condensation to form a gel, which is then calcined to produce oxides [13]. The use of complexing agents (e.g., citric acid, ethylene glycol) enables precise control over particle size and composition, resulting in highly pure and uniform nanoparticles.
3. Hydrothermal or solvothermal method: A precursor solution is heated in a Teflon-lined autoclave at 150–200 °C for 6–24 h, promoting crystallization without the need for high-temperature calcination. This method yields crystalline nanoparticles with well-defined morphology and tunable size [14]. CeO_2_ NPs with an average size of 14 ± 3 nm were synthesized by a hydrothermal method using cerium (III) nitrate hexahydrate as the starting material [15].
4. Combustion or flame synthesis: Rare-earth nitrates react with fuels such as urea or glycine, rapidly forming oxide nanoparticles [16]. This energy-efficient process produces highly porous particles, typically in the 20–30 nm range [17], though calcination may be required to remove residual carbon.
5. Green or biogenic synthesis (eco-friendly). This method uses plant extracts, bacteria, or polysaccharides as reducing/stabilizing agents [18]. Plants are the most efficient source for the green synthesis of nanoparticles because they are a rich source of reducing and stabilizing agents such as phenols, ketones, ascorbic acid, and carboxylic acids. As an example, RE nitrate is mixed with tea, aloe vera, or bacterial extract and heated to form nanoparticles. Biomolecules from the above reducing/stabilizing agents will cap and stabilize particles for controlling aggregation. Biocompatible nanoparticles are suitable for antimicrobial/medical applications. For the preparation of CeO_2_ NPs, cerium nitrate hexahydrate was added to the *Moringa oleifera* leaf extract [19]. The average size of the CeO_2_ NPs was 17 nm, comparable with that of CeO_2_ NPs synthesized using the leaf extracts of *Origanum majorana* (20 nm) [20]. CeO_2_ NPs have been synthesized from Ce(NO_3_)_3_ and seeds extract of *Salvia macrosiphon Boiss* [21]; flower extract of *Calotropis procera* (a particle size of 21 nm) [22]; *Ziziphus jujube* fruit (18–25 nm) [23]; the aqueous extract of *Acorus calamus* rhizome [24]; *Aloe barbadensis* miller gel [25], *Hibiscus sabdariffa* flower [26], *Moringa oleifera* peel [27], *Hyphaene thebaica* fruit [28], citrus limon peel [29], and *Linum usitatissimum* L. seeds [30].

Spherical-shaped CeO_2_ NPs with an average crystallite size of about 8 nm have been synthesized by the extracellular supernatant of *B. subtilis* [31]. Similarly, spherical-shaped CeO_2_ NPs with an average size of 24.5 nm were synthesized by the filtrate of *Fusarium solani* species culture media [32]. The *Aspergillus niger* culture filtrate was used to synthesize CeO_2_ NPs with particle sizes ranging from 5 to 20 nm [33].

In addition to laboratory-scale synthesis routes, a scalable roll-to-roll Sono chemical coating platform has recently been demonstrated for depositing rare-earth oxide nanoparticles onto fabrics and other flexible substrates. The method relies on acoustic cavitation to nucleate and anchor nanoparticles directly on the fiber surface, resulting in unusually strong particle–substrate adhesion. Industrial trials have shown that the coated fabrics retain ≈80% of their nanoparticle loading even after repeated wash cycles at room and elevated temperatures, and continuous coating has been operated for hours on an industrial prototype machine. The scalability and durability of this approach illustrate that sonochemistry can serve not only as a nanoparticle synthesis method but also as a direct surface-functionalization technology for REO-based antibacterial and antiviral materials [34,35,36]. Advantages and challenges to produce REE oxide nanoparticles are summarized in Table 4.

## 4. Diameter and Crystallinity

The average diameters of rare-earth oxide nanoparticles (REO-NPs) depend strongly on the synthesis method, precursor chemistry, and post-treatment (especially calcination temperature). Elevated calcination temperatures lead to increased crystallite size, while higher precursor concentrations result in larger particle formation due to accelerated nucleation rates.

For the precipitation method, higher pH produces smaller, more uniform NPs due to rapid nucleation. Ethanol–water mixtures yield smaller NPs than pure water and the combustion temperature affect crystallite growth (the combustion method). The use of capping agents such as citric acid, PVP, PEG, etc., prevents aggregation and results in smaller, monodisperse particles. Most rare-earth oxide nanoparticles prepared by wet-chemical or sol–gel routes fall in the 5–20 nm range, while higher-temperature or green synthesis routes can produce 20–60 nm particles. A careful control of calcination, pH, and complexing agents allows fine size tuning.

Crystallinity is a crucial parameter for rare-earth oxide (REEO) nanoparticles because it directly affects their surface reactivity, defect density, catalytic and antimicrobial behavior, and cytotoxicity. Table 5 shows a detailed overview of crystallinity characteristics for representative REE oxides, including structure type, typical crystallite size, and implications for bioapplications.

## 5. Antimicrobial Properties of REE-NPS

In view of such combined antimicrobial mechanisms, it is almost impossible for pathogens to develop resistance against the attack of REEs due to the multiple, simultaneous mechanisms of action employed by nanoparticles. Within this scenario, REEs could be used alone or together with antibiotics to combat multidrug resistant (MDR) bacteria.

### 5.1. CeO_2_ NPs

Spherical CeO_2_ NPs with an average size of 17 nm were synthesized by a green, eco-friendly method of “rapid precipitation”, using *Moringa oleifera* leaf extract as a natural reducing and stabilizing agent [19]. The synthesized CeO_2_ NPs demonstrated high antimicrobial activity against *S. aureus*, *E. coli*, and *P. aeruginosa*, as well as antifungal activity against *C. albicans* and *A. fumigatus* [19]. Based on an inhibition zone ranging from 15 to 31 mm, this study suggests potential applications of CeO_2_ NPs as antimicrobial agents.

CeO_2_ NPs with an average size of 22.03 nm synthesized using *Acorus calamus* extract [24] show their antibiofilm activity against *S. aureus* and *P. aeruginosa*. CeO_2_ NPs exhibited significant inhibition zones in disk diffusion assays. The biofilms of the test bacteria were inhibited by more than 75% by the treatment with CeO_2_ -NPs. Biofilm assays (crystal violet method) showed marked biofilm disruption and inhibition at increasing NP concentrations. The mechanism was attributed to electrostatic interaction between positively charged CeO_2_ surfaces and bacterial cell walls, and the release of ^•^OH and O_2_^•−^ to damage bacterial membranes and EPS matrix.

CeO_2_ NPs with an average size of 3.8 nm were synthesized by the hydrothermal method and tested against the selected ESKAPE pathogens [45]. Using agar-well diffusion with 50 µg/mL CeO_2_ NPs, the inhibition zone ranged from 7.53 ± 0.30 mm to 22.25 ± 0.13 mm: *S*. *aureus* 19.40 ± 0.05, *P. aeruginosa* 17.83 ± 0.21, *K. pneumoniae* 17.45 ± 0.01, and *A. baumannii* 8.86 ± 0.03.

CeO_2_ NPs were synthesized by wet chemical/oxalate (hydrolysis/thermal) and tested against four pathogens [46]. Based on a paper disk assay with 50 µg/mL of CeO_2_ NPs, the reported ZOI values (mm) were *E. coli* = 9 ± 0.05, *S. typhimurium* = 12 ± 0.02, *L. monocytogenes* = 10 ± 0.04, *S. aureus =* 5 ± 0.02, and *B. cereus* = 7 ± 0.05.

Green/biosynthesized CeO_2_ NPs (17 nm) were tested against three bacteria and two fungi [47]. The inhibition zones of CeO_2_ NP (100 mg/mL) antibacterial test against *S. aureus*, *P. aeruginosa*, and *E. coli* bacteria were 22, 16, and 15 mm, respectively, compared with 20 mm for *C. albicans* and 26 mm for *A. fumigatus*.

Pure CeO_2_ NPs were produced by pulsed laser ablation in liquid using an Nd:YAG laser [37]. The particles were nearly spherical with an average size of 10–30 nm and a Zeta potential around –25 mV, indicating moderate colloidal stability. Antibacterial activity increased with nanoparticle concentration. At 100 µg/mL, they exhibited antibacterial activities against *E. coli* and *S. aureus* with an inhibition zone diameter of 16 ± 0.4 mm and 14 ± 0.3 mm, respectively. The killing mechanism was attributed to ROS generation (O_2_^•−^ and H_2_O_2_) to cause bacterial membrane disruption.

The hybrid chitosan CeO_2_ NPs were prepared using plant leaf extract [38]. The formation of Ce-O bonds and chitosan main chains were affected by the COC and CO groups. The prepared hybrid chitosan-CeO_2_ NPs were predominantly uniform in size, spherical with particles size ranging from 3.61 nm to 24.40 nm. The Ce^3+^/Ce^4+^ redox cycle at the nanoparticle surface generated ROS such as superoxide radicals (O_2_^−•^) and hydroxyl radicals (^•^OH) to damage cell membranes, proteins, and DNA in bacteria. Chitosan carries positive amino groups (–NH_3_^+^) that interact with negatively charged bacterial membranes, increasing membrane permeability and leakage of intracellular contents. Chitosan helps in the uniform dispersion of CeO_2_ NPs and enhances cell adhesion, while CeO_2_ catalyzes oxidative stress that kills the bacteria. Quantitative inhibition zone data shows the antibacterial efficacy of the hybrid chitosan–CeO_2_ nanoparticles compared with chitosan alone and pure CeO_2_ nanoparticles (Table 6).

The antibacterial effect of cerium oxide nanoparticles (CeO_2_ NPs) and CeO_2_ NPs incorporated into nanofiber scaffolds (Scaffold@ CeO_2_ NPs) against *P. aeruginosa* was assessed [48]. The MIC (Minimum Inhibitory Concentrations) for bare CeO_2_ NPs was 12.5 µg/mL and 6.25 µg/mL for Scaffold@ CeO_2_ NPs, while MBC (Minimum Bactericidal Concentration) values were 50 µg/mL for both. At the highest concentration of CeO_2_ NPs in the scaffold (200 µg/mL), 97% of human fibroblast cells survived, indicating low toxicity at concentrations effective for bacterial inhibition.

The antimicrobial activity of nanocrystalline CeO_2_ was evaluated against Pseudomonas aeruginosa, a common hospital-acquired pathogen [49]. This activity is primarily attributed to the physicochemical properties of CeO_2_ nanoparticles, specifically the presence of oxygen vacancies and the ability of cerium ions to alternate between Ce^3+^ and Ce^4+^ oxidation states. This redox cycle promotes the generation of reactive oxygen species (ROS), which damage bacterial components and disrupt essential processes such as respiration and DNA replication. At an optimal concentration of 10^−3^ M, Nanoceria inhibited *E. coli* growth by 48–95% after 24 to 48 h of incubation, depending on the concentration used.

The antimicrobial activity of cerium oxide nanoparticles (CeO_2_ NPs) against periodontal pathogens demonstrated a dose-dependent reduction in the proliferation of *Porphyromonas gingivalis* and *Prevotella intermedia* [50]. A 2 mg/mL concentration of a 5% CeO_2_ sample reduced *P. gingivalis* cells by 94.6% ± 7.7% but only reduced *P. intermedia* by 70% ± 0.8%. CeO_2_ NPs are biocompatible with human periodontal ligament cells, promote osteogenic differentiation and cell proliferation, and exhibit antimicrobial properties, particularly against *P. gingivalis*. They could be an excellent choice for use in clinical practices, such as in dental implant surface modifications.

Cerium dioxide-dextran nanocomposites exhibit a bacteriostatic effect against *E. coli* [51]. The formulation (cerium nitrate to dextran ratio 1:2), at a concentration of 10^−3^ M, reduces the rate of microorganism multiplication by three to four times. After 48 h of incubation, all tested nanocomposite formulations suppressed the multiplication of *E. coli* by 58–77% compared to the control group. The dose-dependent effect was also confirmed, as after 48 h, *E. coli* multiplication was inhibited by 47% at 10^−5^ M and up to 80% at 10^−2^ M. This antimicrobial effect is a key characteristic for fostering nanocomposites into a medical product for wound healing.

### 5.2. Er_2_O_3_ NPs

An ionic liquid, 1-butyl-3-methylimidazolium hexafluorophosphate (BMIM-PF_6_) (“BMIM-PF_6_ IL”) serves as a templating/capping agent in the polyol synthesis of Er_2_O_3_ NPs [52]. The IL-Er_2_O_3_ NPs achieved inhibition zones of ~12 mm for *S. aureus* and ~14 mm for *E. coli* at a concentration of 25 µg/mL. It was proposed that IL-mediated NPs generate more oxygen vacancies (defects), which in turn help produce reactive oxygen species (ROS) like singlet oxygen (^1^O_2_), superoxide (O_2_), and hydroxyl radicals (OH). Er_2_O_3_ NPs bind to the cell surface via van der Waals interactions to disrupt bacterial membranes and cause the generation of intracellular ROS to damage mitochondrial DNA, DNA sugar backbone, lipids, and proteins, leading to cell lysis. The increased oxygen vacancies and ROS generation capacity of the IL-NPs contribute to their enhanced bioactivity.

The antibacterial properties and aquatic toxicity of three rare-earth oxide nanoparticles (REOs), Gd_2_O_3_, Sm_2_O_3_, and Er_2_O_3_, have been investigated [40] to assess whether these nanoparticles exhibit antibacterial activity under daylight illumination (photoinduced effect). Under daylight, the nanoparticles generated reactive oxygen species (ROS), which caused oxidative stress and bacterial cell damage. In the dark, antibacterial activity was negligible or minimal. Gd_2_O_3_ showed the strongest antibacterial effect, followed by Sm_2_O_3_, then Er_2_O_3_. The lowest MICs were 5.6 mg mL^−1^ against *E. coli* and *P. aeruginosa*, which is high compared to other strong antimicrobial agents.

### 5.3. Eu_2_O_3_ NPs

Eu_2_O_3_ nanoparticles were mixed into methyl cellulose (MC) solutions at several weight percentages (0.0, 0.50, 0.75, 1.00, 1.25, and 1.50 wt%) and cast as films. MC films loaded with europium oxide (Eu_2_O_3_) nanoparticles reduced the survival and attachment of common foodborne bacteria (*E. coli*, *S. typhimurium*, and *S. aureus*) and so could be useful as antimicrobial food-packaging films. MC films doped with Eu_2_O_3_ produced significantly lower viable counts of *E. coli*, *S. typhimurium,* and *S. aureus* compared with the pure MC film [53]. The attachment/adherence assays indicated weaker bacterial attachment to Eu_2_O_3_-doped films vs. plain MC. Antibacterial efficacy rose nearly linearly with increasing Eu_2_O_3_ nanoparticle loading. The 1.25–1.50 wt% films achieved ~3 log reductions (99.9%) in viable counts of *Salmonella* and *S. aureus*, and nearly 3 log for *E. coli*. Adherence/attachment of all three pathogens dropped by ~50–60% at the highest doping level, confirming reduced bacterial colonization of the film surfaces.

### 5.4. La_2_O_3_ NPs

The green synthesis of lanthanum oxide nanoparticles (La_2_O_3_ NPs) can be performed using *Couroupita guianensis* (Cannonball tree) leaf extract as a natural reducing and stabilizing agent [54]. Aqueous leaf extract was simply mixed with lanthanum nitrate solution and heated to form La_2_O_3_ NPs with an average size of ~20–30 nm. The particles were capped with phenols, flavonoids, and carboxylic acids. The La_2_O_3_ NPs exhibited noticeable zones of inhibition (ZOI), approximately 14–18 mm *E. coli* and *S. aureus* using the agar well diffusion method. The antibacterial effect was attributed to reactive oxygen species (ROS) generation and interaction with bacterial membranes.

La_2_O_3_ NPs (size: ~35 nm) were evaluated for their effects on the growth, lipid accumulation, oxidative stress, and cell integrity of the biodiesel-related fungus *Moniliella wahieum* Y12T [55]. La_2_O_3_ NPs exhibited strong, concentration-dependent antifungal toxicity against *Moniliella wahieum* Y12T. Even at 50 mg/L, La_2_O_3_ NPs disrupted growth (~25% reduction) and lipid accumulation (~15% reduction) through oxidative stress and membrane damage.

### 5.5. Nd_2_O_3_ NPs

Nd_2_O_3_ nanoparticles synthesized via ionic liquid–assisted green methods using *Couroupita guianensis* leaf extract are biocompatible and highly pure, with a crystallite size of 25–30 nm [56]. They show strong antimicrobial activity: *S. aureus* (ZOI: 15 mm, MIC: 100 µg/mL), *E. coli* (17 mm, 75 µg/mL), *P. aeruginosa* (13 mm, 125 µg/mL), and *C. albicans* (12 mm, 100 µg/mL). The mechanism involves ROS generation and ionic disruption, damaging microbial membranes and DNA. Superoxide and hydroxyl radicals damage membranes and DNA. Nd_2_O_3_ NPs adsorb electrostatically onto bacterial cell surfaces. Nd^3+^ ions may displace essential divalent cations, disrupting metabolic processes. Nd_2_O_3_ is especially effective against Gram-negative bacteria due to easier cell wall penetration.

### 5.6. Pr_2_O_3_ NPs

The study evaluated the toxicity and biocompatibility of bare praseodymium oxide nanoparticles (Pr_2_O_3_ NPs) and those functionalized with non-ionic surfactants (Tween-20, Tween-80, and Triton X-100) [3]. Cytotoxicity (in vitro) was tested on L929 fibroblast and HEK293 kidney cell lines. In vivo toxicity was tested on a zebrafish embryo model. Surface modification with non-ionic surfactants reduced Pr_2_O_3_ NP toxicity by enhancing stability and lowering oxidative stress. Functionalized Pr_2_O_3_ NPs may be biocompatible for biomedical and environmental applications if properly surface treated. Toxic effects are mainly mediated through ROS generation and oxidative stress pathways. Toxicity ranking: Bare > Triton X-100- > Tween-20- > Tween-80-functionalized NPs.

### 5.7. Sc_2_O_3_ NPs

Electrospun PCL/PVP nanofiber membranes were loaded with nano-textured Sc_2_O_3_-MgO (0–3 wt% loadings) [57]. With 2.0 wt% loading, they achieved smooth, bead-free fibers (avg diameter ~250 nm) and achieved complete inhibition of *E. coli* under their test conditions (100% antibacterial rate). The composites also show acceptable preliminary biosafety. The study suggests promises for antibacterial wound dressings or tissue engineering scaffolds, though further work is needed to elucidate the mechanism and bacterial spectrum, in vivo testing, and longer-term biocompatibility. Sc_2_O_3_–MgO/PCL/PVP membranes exhibit the highest antibacterial performance (100%) at a relatively low oxide loading (2 wt%)—outperforming most other single-oxide systems. Hybrid polymer matrices (like chitosan, PCL, and PVP) generally enhance dispersion and surface contact, amplifying oxide activity. Overall, the Sc-MgO system achieves strong inhibition while maintaining fibrous biocompatibility and structural stability, positioning it as a next-generation antibacterial dressing candidate.

### 5.8. Sm_2_O_3_ NPs

Samarium oxide nanoparticles (Sm_2_O_3_ NPs) were synthesized and their effects on biofilm, virulence factors, and motility of MDR *P. aeruginosa* were evaluated [58]. The Sm_2_O_3_ nanoparticles were green synthesized using curcumin as a reducing/stabilizing agent. The average particle size is ≈20 nm and Sm_2_O_3_ NPs have an MIC of 250 µg/mL and inhibit biofilm formation (49–61% reduction); pyocyanin synthesis (33–55% reduction), protease production (24–45% reduction), hemolytic activity (2–41% reduction), and motility (reduction 40–60%). Sub-MIC levels could be ≤125 µg/mL, indicating that Sm_2_O_3_ NPs act mainly as anti-virulence and anti-biofilm agents rather than direct bactericidal materials at lower concentrations, suggesting a quorum-sensing-interference mechanism.

Sm_2_O_3_ NPs were synthesized via a green hydrothermal method using the leaf extract of *Andrographis paniculata* plus an ionic liquid, 1-butyl-3-methylimidazolium hexafluorophosphate (BMIM PF_6_), as a capping/stabilizing agent [59]. The particles exhibited body-centered cubic structure with an average particle size of about 30–50 nm. The plant extract provides reducing/stabilizing phytochemicals; the ionic liquid helps control morphology and stability. The IL-assisted Sm_2_O_3_ NPs (IL-Sm_2_O_3_) showed a higher inhibition rate in *E. coli* than *S. aureus* and this result was somewhat unexpected as Gram-negative bacteria are more resistant to antibiotics.

Gram-positive bacteria have a thick peptidoglycan cell wall and a single cell membrane, retaining crystal violet to appear purple; Gram-negative bacteria have a thin peptidoglycan layer plus an outer lipid membrane containing lipopolysaccharides (LPS) and porins, appearing pink/red after staining due to the outer membrane blocking the primary stain. This extra outer membrane makes Gram-negative bacteria more resistant to antibiotics.

The IL-assisted Sm_2_O_3_ NPs exhibited a higher inhibition rate to the standard amikacin against *E. coli* and *S. aureus* bacterial systems. The killing mechanism was attributed to the generation of ROS to cause oxidative stress in bacteria, whereas cationic Sm^3+^ interactions damaged DNA and cell membranes.

Of note is the green synthesis of samarium oxide/silver core–shell nanoparticles (Sm_2_O_3_@Ag NPs) using onion peel waste extract as a reducing and stabilizing agent, aiming to develop an eco-friendly material with enhanced antimicrobial activity [60]. Samarium nitrate was first converted to Sm_2_O_3_ nanoparticles via thermal treatment, followed by Ag shell deposition using silver nitrate reduced by onion peel phytochemicals. The particles with an average particle size of 25–35 nm had a crystalline cubic Sm_2_O_3_ core and a face-centered cubic Ag shell with functional groups (–OH, C=O, phenolics). Sm_2_O_3_@Ag NPs exhibited significantly larger ZOIs (25–35 mm) compared to bare Sm_2_O_3_ (12–16 mm) or Ag NPs alone (~20 mm) for all tested bacteria: *Staphylococcus aureus*, *B. subtilis*, *E. coli*, and *P. aeruginosa*. The MIC values range between 12.5 and 25 µg/mL, depending on the strain. Their antifungal activity was notable against *C. albicans* and *A. niger* with a ZOI up to 28 mm. The core–shell structure demonstrated synergistic antibacterial effects, likely due to reactive oxygen species (ROS) generation and silver ion release.

### 5.9. Tb_4_O_7_ NPs

Commercial Tb_4_O_7_ (terbium oxide) NPs have a hydrodynamic size ~400 nm, core size ~200 nm), and a zeta potential ~ +31.6 mV in water [61]. Tb_4_O_7_ NPs could oxidize ascorbic acid (AA) (a bacterial antioxidant) and generate H_2_O_2_ in situ; further, they catalyzed H_2_O_2_ → OH. In vitro, Tb_4_O_7_ NPs showed concentration-dependent killing of bacteria: *S. aureus* at 100 µg/mL were about ~90% killed, and *E. coli* showed a similar trend. Intracellular ROS levels in bacteria increased significantly with NP treatment (DCFH-DA fluorescence). In vivo wound healing: In the treated mice, wounds shrank faster: by day 7, wounds treated with Tb_4_O_7_ NPs were nearly healed, whereas the control still had an obvious scab. Bacterial numbers in wound tissue on day 7 were significantly lower in NP-treated vs. control mice.

### 5.10. Tm_2_O_3_ NPs

Thulium nanoparticles (Tm_2_O_3_ NPs) are represented by oxide and hexacyanoferrate II. Thulium oxide (Tm_2_O_3_) nanoparticles were synthesized and tested ROS generation under ultrasound and antibacterial activity vs. common bacteria [62]. Ultrasound triggered ROS production and measurable antibacterial activity. NaYF_4_: Yb,Tm upconversion nanoparticles (UCNPs) were prepared and coated/assembled with a poly(selenoviologen) photosensitizer to produce both ROS (PDT) and heat (PTT) under 980 nm NIR and tested in vitro and in vivo against MRSA, showing high efficiency in killing MRSA both in vitro and in vivo [63]. β-NaYF_4_:Yb,Tm UCNPs were prepared and converted to β-NaYF_4_:Yb,Tm@ZnO to use NIR→UV upconversion to activate ZnO and generate ROS and were tested against *S. aureus* and *S. aureus* small colony variants (SCV). NIR-activated UCNP@ZnO produced 0.67 log CFU reductions in *S. aureus* (WCH-SK2) and 0.76 log CFU reductions in the SCV formed under their test conditions [64]. NaYF_4_:Yb,Tm-based UCNPs were built and coupled to TiO_2_/Ag_3_PO_4_ photocatalysts to extend visible/NIR response and were tested on their photocatalytic degradation of pollutants and pathogen inactivation [65].

There is a comprehensive review of UCNP platforms (including Tm-doped systems like NaYF_4_:Yb,Tm) used to drive photodynamic/photothermal antibacterial therapies, mechanisms (NIR→UV/vis upconversion to activate photosensitizers or photocatalysts), and typical reported outcomes (inactivation percentages, log-CFU reductions, in vitro and some in vivo results) [66]. The review compiles many studies and reports typical bacterial inactivation results ranging from modest log reductions (0.5–2 log) up to near-complete inactivation depending on the UCNP hybrid, light dose, and photosensitizer; exact numbers depend highly on material and conditions. 

## 6. Cytotoxicity, In Vivo Safety, Pharmacokinetics, and Long-Term Biological Effects

Rare-earth oxide nanoparticles (REO-NPs) exhibit dose-dependent cytotoxicity that varies with composition, particle size, surface chemistry, and cell type. Smaller particles (<10 nm), uncoated oxides, and high-defect surfaces tend to generate more reactive oxygen species (ROS), contributing to mitochondrial dysfunction, membrane damage, and apoptosis. At moderate sizes (15–50 nm), most REO-NPs—including CeO_2_, La_2_O_3_, Sm_2_O_3_, Nd_2_O_3_, and Gd_2_O_3_—show relatively low cytotoxicity toward fibroblasts and epithelial cells at ≤100 µg/mL. Surface functionalization (PEG, PVP, polysaccharides, and chitosan) consistently reduces oxidative reactivity and enhances biocompatibility by limiting ion dissolution and stabilizing surface charge.

Cytotoxicity values (IC_50_) for REO-NPs typically range between 50 and 300 µg/mL depending on the oxide and cell line. CeO_2_ exhibits the most favorable cytotoxicity profile due to its ability to alternate between Ce^3+^ and Ce^4+^, allowing it to act as either a ROS generator in bacteria or a ROS scavenger in mammalian cells. Heavier REOs such as Er_2_O_3_ or Tm_2_O_3_ tend to induce more oxidative stress at equivalent doses. The values of IC_50_ are inversely related to toxicity for versus cell lines, as listed in Table 7.

### 6.1. In Vivo Biodistribution, Clearance, and Long-Term Safety

Following systemic administration, REO-NPs generally accumulate in the liver and spleen due to uptake by mononuclear phagocyte system macrophages [74]. Larger particles (>15–20 nm) undergo slow hepatobiliary clearance and may persist for weeks, whereas particles approaching the renal filtration threshold (<8–10 nm) demonstrate partial urinary excretion [75]. Dissolved RE^3+^ ions may be complex with endogenous phosphates, influencing their long-term fate and reducing free-ion toxicity. Surface coatings (PEGylation and protein coronas) extend circulation time but reduce nonspecific organ uptake and reactivity [76].

In vivo toxicology studies indicate that REO-NPs are well-tolerated at therapeutic antimicrobial doses, although chronic exposure can elevate liver enzymes, inflammatory cytokines, and oxidative biomarkers [77]. La_2_O_3_ and Gd_2_O_3_ may exhibit higher organ-level reactivity due to ion release. Long-term safety data remain limited, and comprehensive toxicology—including genotoxicity, immune activation, and biodegradation—is required for regulatory consideration.

### 6.2. Stability and Aggregation in Biological Media

Colloidal stability is a critical determinant of antimicrobial efficacy and biological interactions. In physiological fluids, electrolytes compress the electrical double layer and promote aggregation [78]. Proteins such as albumin, fibrinogen, and wound exudate components readily form coronas around REO-NPs, altering surface charge and blocking catalytic redox sites. Aggregation decreases oxygen-vacancy accessibility and reduces ROS-dependent antimicrobial activity.

Surface modification provides a means of controlling stability:

- PEG/PVP coatings improve colloidal stability but may mask active surface sites.
- Chitosan/polysaccharide coatings enhance bacterial adhesion and improve antimicrobial effects, though they may compromise long-term dispersion stability.
- Metal dopants (Ag, Zn, and Cu) increase antimicrobial potency but may raise cytotoxicity concerns.

For antimicrobial applications—especially in chronic wound environments—REO-NPs require stable dispersion for at least 24–48 h under high-salt, protein-rich conditions [79]. Optimal stability is associated with zeta potentials |ζ| ≥ 25 mV, minimal corona formation, and controlled Ce^3+^/Ce^4+^ surface chemistry.

### 6.3. Industrial Scalability, GMP Manufacturing, and Environmental Considerations

Most laboratory synthesis methods including sol–gel, hydrothermal, precipitation, and green biogenic routes produce high-quality REO-NPs but are not directly scalable. Green synthesis offers improved biocompatibility but suffers from batch-to-batch chemical variability. Hydrothermal and solvothermal methods are energy-intensive and typically limited to small-batch production. Sol–gel approaches require high-temperature calcination and produce solvent waste.

Emerging strategies for industrial-scale manufacturing include the following:

- Continuous-flow reactors that ensure uniform nucleation and narrow size distribution.
- Microwave-assisted synthesis, enabling rapid crystallization with reduced energy input.
- Spray pyrolysis and flame spray pyrolysis, which offer kilogram-scale production for medical-grade powders
- Sono-chemical roll-to-roll coating, which enables direct deposition on wound dressings and textiles.

For clinical use, Good Manufacturing Practice (GMP) requirements include endotoxin-free production, closed-system processing, batch documentation, and stringent control of particle size, crystallinity, and surface chemistry. Long-term stability and toxicology data must accompany each production lot.

Environmental considerations remain important: REE extraction is energy-intensive and may produce acidic and fluorinated waste streams. Sustainable manufacturing demands hydrometallurgical recovery, reduced reagent consumption, solvent recycling, and energy-efficient reactors.

## 7. Trends and Future Directions

Rare-earth oxide nanoparticles (REO-NPs) exhibit strong antimicrobial activity against Gram-negative and Gram-positive bacteria through multifactorial mechanisms, including ROS generation, membrane disruption, and ion exchange. Antibacterial efficacy generally improves with smaller particle size, higher surface oxygen-vacancy density, and optimized surface charge. CeO_2_ remains the most promising REE oxide due to its favorable balance of antibacterial performance and mammalian biocompatibility. Most REO-NPs achieve meaningful antimicrobial effects at concentrations of 25–75 µg/mL, while higher doses may increase cytotoxicity depending on composition, size, and surface chemistry.

For future development, improved surface functionalization (e.g., chitosan, PEG, and citrate) will be crucial for enhancing antimicrobial selectivity while minimizing mammalian cell toxicity. Hybrid structures—such as CeO_2_@chitosan or La_2_O_3_-based composites—offer synergistic antibacterial responses and may overcome limitations of individual oxides. Standardized MIC/ZOI testing, cytotoxicity protocols, and long-term in vivo safety assessments are needed to ensure comparability across studies and to guide translational development.

Scalability remains a major barrier. Laboratory synthesis methods such as sol–gel, hydrothermal, and green biogenic routes provide excellent control of size and morphology but are difficult to scale to industrial volumes. Continuous-flow microreactor synthesis, spray pyrolysis, and microwave-assisted crystallization represent realistic routes to industrial-scale production with improved reproducibility. For clinical translation, GMP manufacturing will require endotoxin-free processes, closed reactors, and strict control of particle size distribution, crystallinity, and surface chemistry.

Environmental sustainability also remains a challenge. REE extraction and oxide synthesis are energy-intensive and may generate acidic and fluorinated waste streams. A combination of cleaner hydrometallurgical recovery reduced solvent consumption, and energy-efficient reactors will be necessary to minimize environmental impacts. Overall, REO-NPs hold considerable promises for antimicrobial coatings, wound dressings, implant surface modification, and catalytic disinfection platforms. Advancements in scalable synthesis, surface engineering, and comprehensive toxicological evaluation will be pivotal in enabling their safe and effective integration into biomedical applications.

## 8. Conclusions

For the synthesis of rare-earth element oxides, future trends will focus on the development of continuous-flow reactors, which are scalable and reproducible under green conditions. Bioreactor-based microbial synthesis (yeast, fungi, and cyanobacteria) is still experimental but promising for green scale-up. The recovery of REEs from electronic waste for sustainable nanoparticle production might be considered. The development of hybrid composites for synergistic effects and surface functionalization of rare-earth oxides is needed to enhance selectivity and reduce cytotoxicity. Rare-earth oxide nanoparticles represent a new frontier in the fight against multidrug-resistant pathogens. To fully realize the clinical potential of rare-earth oxide nanoparticles, it is essential to address toxicity, scalability of production, and long-term biosafety. Key biomedical applications include antimicrobial coatings, wound dressings, and hybrid nanozyme systems for oxidative disinfection.

## Figures and Tables

**Table 1:** Seventeen rare-earth metals, common oxidation state, and key applications.

Atomic Number, Element, Symbol	Oxidation State	Key Applications
21 Scandium (Sc)	+3	Aerospace alloys, sports equipment
39 Yttrium (Y)	+3	LEDs, superconductors, phosphors
57 Lanthanum (La)	+3	Camera lenses, hybrid car batteries
58 Cerium (Ce)	+3/+4	Catalytic converters, glass polishing
59 Praseodymium (Pr)	+3	Magnets, aircraft engines, glass coloring
60 Neodymium (Nd)	+3	Strong magnets (NdFeB), lasers
61 Promethium (Pm)	+3	Radioisotope batteries, research (rare, radioactive)
62 Samarium (Sm)	+3	Samarium–cobalt magnets, nuclear control rods
63 Europium (Eu)	+2/+3	Red and blue phosphors in TV and LED screens
64 Gadolinium (Gd)	+3	MRI contrast agents, neutron shields
65 Terbium (Tb)	+3	Green phosphors, solid-state devices
66 Dysprosium (Dy)	+3	Magnets, laser materials, nuclear reactors
67 Holmium (Ho)	+3	Lasers, magnets
68 Erbium (Er)	+3	Optical fibers, lasers, glass tinting
69 Thulium (Tm)	+3	X-ray machines, portable lasers
70 Ytterbium (Yb)	+3	Fiber optics, stress gauges, semiconductors
71 Lutetium (Lu)	+3	PET scan detectors, catalysts

**Table 2:** Key properties of the rare-earth elements (REE) with important properties for medical/biomedical applications [4].

RRE	Antitumor	Antioxidant	Antidiabetic	Contrasting Agent	Regenerative
Cerium (Ce)	X	X			X (X = positive)
Dysprosium (Dy)	X			X	
Erbium (Er)	X		X	X	
Europium (Eu)	X			X	X
Gadolinium (Gd)	X			X	X
Holmium (Ho)	X	X	X		
Lanthanum (La)	X	X			
Lutetium (Lu)	X				
Neodymium (Nd)	X	X	X		
Praseodymium (Pr)	X	X			
Promethium (Pm)	N/A	N/A	N/A	N/A (not available)	N/A
Samarium (Sm)	X	X	X		
Scandium (Sc)			X		
Terbium (Tb)	X	X	X		
Thulium (Tm)	X				
Ytterbium (Yb)	X	X	X		
Yttrium (Y)	X	X	X		

**Table 3:** The primary effects of some selected REEs and their oxide nanoparticles against pathogens.

Mechanism	Primary Effect Involved	Key Elements
Membrane disruption	Leakage, lysis	La^3+^, Ce^3+^, Gd^3+^
Ion substitution	Enzyme/membrane dysfunction	La^3+^, Y^3+^, Nd^3+^
ROS generation	Oxidative stress	Ce^3+^/Ce^4+^
DNA/protein binding	Replication/enzyme inhibition	La^3+^, Eu^3+^
Enzyme inhibition	Metabolic arrest	Gd^3+^, Y^3+^
Biofilm interference	Anti-quorum sensing	La^3+^, Ce^3+^
Nanoparticle redox effects	Dynamic antimicrobial activity	CeO_2_, La_2_O_3_

**Table 4:** A comparison of different methods to produce REE oxide nanoparticles.

Method	Typical Scale Potential	Advantages	Challenges
Precipitation/Co-precipitation	High (industrial)	Simple, low cost, aqueous	Poor control of morphology, agglomeration
Sol–gel/Pechini	Moderate	Homogeneous mixing, high purity	Organic residue removal, energy-intensive calcination
Spray pyrolysis/Flame spray	Industrial	Continuous, tunable	High energy use, limited for “green” applications
Hydrothermal/Solvothermal	Lab to pilot	Good crystal control	Pressure reactors, batch process
Microemulsion/Reverse micelle	Lab	Fine size control	Costly surfactants, low yield
Green/Biogenic synthesis (plant or microbial mediated)	Low to moderate	Low toxicity, mild conditions	Batch variability, slower, scale-up difficult

**Table 5:** Crystallinity and structural characteristics of rare-earth oxides (REOs).

REOs	Crystal System/Phase	Typical Crystallite Size (nm)	Dominant XRD Peaks	Bioimpact/Cytotoxicity	Refs.
CeO_2_	Cubic (Fm3̅m)	5–50	(111), (200), (220), (311)	High ROS via Ce^3+^/Ce^4+^ redox; strong antibacterial but may induce cytotoxicity at high defect levels.	[37,38]
La_2_O_3_	Hexagonal (P6_3_/mmc) → Monoclinic (C2/m)	10–80	(100), (002), (101)	Low ROS; mild antimicrobial and low cytotoxicity.	[39]
Nd_2_O_3_	Cubic (Ia3̅)/Hexagonal (P6_3_/mmc)	15–60	(222), (400), (440)	Moderate crystallinity; stable, moderate antimicrobial effect.	[40]
Gd_2_O_3_	Cubic (Ia3̅)	5–40	(222), (400), (440)	Highly crystalline; good for MRI and photodynamic therapy; biocompatible at <25 nm.	[41]
Sm_2_O_3_	Cubic (Ia3̅) → Monoclinic (C2/m)	10–50	(222), (400), (440)	Intermediate ROS generation; moderate antimicrobial activity.	[42]
Er_2_O_3_	Cubic (Ia3̅)	8–35	(222), (400), (440)	Stable cubic phase; low cytotoxicity, moderate antimicrobial effect.	[42]
Y_2_O_3_	Cubic (Ia3̅)	10–30	(222), (400), (440)	High stability, inert, low toxicity, used in coatings and tracers.	[43]
Pr_6_O_11_	Mixed cubic + hexagonal	5–25	(200), (220), (311)	Strong ROS producer; potent antibacterial but higher cytotoxicity risk.	[44]

**Table 6:** Inhibition zone diameter (mm) obtained by the agar well diffusion method, 100 µg/mL concentration [38].

Bacterial Strain	Chitosan	CeO_2_ NPs	Chitosan–CeO_2_ NPs
*S. aureus* (Gram +)	10 ± 0.5	12 ± 0.4	18 ± 0.6
*E. coli* (Gram –)	9 ± 0.3	11 ± 0.5	17 ± 0.5
*P. aeruginosa* (Gram –)	8 ± 0.4	10 ± 0.5	15 ± 0.4

**Table 7:** A comparison of the cytotoxicity of different REE oxides.

Oxide	IC_50_/Toxicity µg/mL	Cell Line(s)	Main Observations	Ref.
CeO_2_	~110 (moderate)	HeLa, L929, A549	Generally low cytotoxicity due to Ce^3+^/Ce^4+^ redox cycling and ROS scavenging; sometimes protective at low dose.	[67]
Er_2_O_3_	3.2 (high)	L929 fibroblasts	Pronounced toxicity at low concentration; ROS generation implicated.	[40]
Eu_2_O_3_	≈50–80 (moderate)	HepG2, HeLa	Moderate oxidative stress, mitochondrial damage at higher doses.	[68]
Gd_2_O_3_	304 (low)	A549, HepG2	Low toxicity; internalized slowly; used safely as MRI contrast precursor.	[69]
La_2_O_3_	300 (low)	L929, A549	Minimal cytotoxicity below 200 µg/mL; surface hydroxylation reduces reactivity.	[70]
Nd_2_O_3_	~200 (low)	MCF-7, A549	Moderate toxicity: ionic dissolution contributes to stress.	[71]
Pr_6_O_11_	>250 (low)	L929	Very low cytotoxicity; surface inertness limits interaction.	[3]
Sm_2_O_3_	>200 (low)	L929	Comparable to La_2_O_3_; minimal oxidative effect.	[40,58]
Sc_2_O_3_	~180 (low–moderate)	NIH-3T3	Generally biocompatible but may produce ROS under UV illumination.	[57]
Tb_4_O_7_	~90 (moderate)	HepG2	Mild cytotoxicity; oxidative stress at high concentrations.	[72]
Tm_2_O_3_	~150 (low–moderate)	HeLa	Mild toxicity; photoluminescent but stable.	[73]
